# How mycobacterium tuberculosis infection could lead to the increasing risks of chronic fatigue syndrome and the potential immunological effects: a population-based retrospective cohort study

**DOI:** 10.1186/s12967-022-03301-1

**Published:** 2022-02-21

**Authors:** Tse-Yen Yang, Cheng-Li Lin, Wei-Cheng Yao, Chon-Fu Lio, Wen-Po Chiang, Kuan Lin, Chien-Feng Kuo, Shin-Yi Tsai

**Affiliations:** 1 Molecular and Genomic Epidemiology Center, China Medical University Hospital, Taichung City, 404 Taiwan; 2 College of Medicine, China Medical University, Taichung City, 404 Taiwan; 3 Management Office for Health Data, China Medical University Hospital, Taichung City, 404 Taiwan; 4 Department of Anesthesiology and Pain Medicine, Min-Sheng General Hospital, Tao-Yuan City, 330 Taiwan; 5 Department of Laboratory Medicine, Mackay Memorial Hospital, Taipei City, 104 Taiwan; 6 Department of Medicine, Mackay Medical College, New Taipei City, 252 Taiwan; 7 Institute of Infectious Disease, Mackay Memorial Hospital, Taipei City, 104 Taiwan; 8 Graduate Institute of Long-Term Care, Mackay Medical College, New Taipei City, 252 Taiwan; 9 Graduate Institute of Biomedical Sciences, Mackay Medical College, New Taipei City, 252 Taiwan; 10 Department of Health Policy and Management, Johns Hopkins University Bloomberg School of Public Health, Baltimore, 21205 USA

**Keywords:** Mycobacterium tuberculosis infection (MTI), Chronic fatigue syndrome (CFS), Immunological effect, Risk factors, Population-based retrospective cohort study

## Abstract

**Background:**

Chronic fatigue syndrome (CFS) has been shown to be associated with infections. Tuberculosis (TB) is a highly prevalent infectious disease. Patients with chronic fatigue syndrome and post-tuberculosis experience similar symptoms. Furthermore, chronic fatigue syndrome and tuberculosis share similar plasma immunosignatures. This study aimed to clarify the risk of chronic fatigue syndrome following the diagnosis of Mycobacterium tuberculosis infection (MTI), by analyzing the National Health Insurance Research Database of Taiwan.

**Methods:**

7666 patients aged 20 years or older with newly diagnosed Mycobacterium tuberculosis infection during 2000–2011 and 30,663 participants without Mycobacterium tuberculosis infection were identified. Both groups were followed up until the diagnoses of chronic fatigue syndrome were made at the end of 2011.

**Results:**

The relationship between Mycobacterium tuberculosis infection and the subsequent risk of chronic fatigue syndrome was estimated through Cox proportional hazards regression analysis, with the incidence density rates being 3.04 and 3.69 per 1000 person‐years among the non‐Mycobacterium tuberculosis infection and Mycobacterium tuberculosis infection populations, respectively (adjusted hazard ratio [HR] = 1.23, with 95% confidence interval [CI] 1.03–1.47). In the stratified analysis, the Mycobacterium tuberculosis infection group were consistently associated with a higher risk of chronic fatigue syndrome in the male sex (HR = 1.27, 95% CI 1.02–1.58) and age group of ≥ 65 years old (HR = 2.50, 95% CI 1.86–3.38).

**Conclusions:**

The data from this population‐based retrospective cohort study revealed that Mycobacterium tuberculosis infection is associated with an elevated risk of subsequent chronic fatigue syndrome.

## Background

Chronic fatigue syndrome (CFS) is conventionally defined as the presence of unexplainable fatigue lasting > 6 months and accompanied by at least four of the following symptoms: substantial impairment in short-term memory, tender lymph nodes, sore throat, muscle pain, multiple joint pain without swelling or redness, headache, unrefreshing sleep, and postexertional malaise lasting > 24 h [1]. CFS affects not only physical but mental status with profound disability. It was considered a psychiatric disorder due to a lack of a consistent physiological marker or physical finding [2, 3]. Psychiatric conditions such as anxiety, sleep disorders, and depression are strongly related to CFS [4, 5]. It can lead to impairment in simple and complex information processing speed and tasks requiring working memory [6], and imposes huge economic costs on society [7]. The etiologies of chronic fatigue syndrome involve multiple factors. Current studies revealed the etiologies are related to infection [8], immune system differences [9], endocrine-metabolic dysfunction [10], or some specific disease such as peptic ulcer disease [11]. Multiple infectious agents have been linked to CFS, such as the varicella zoster virus [9]. Identifying potential patients with CFS from among post-infectious patients is crucial for early diagnosis and prevention. With every third person on the Earth having *Mycobacterium tuberculosis* infection (MTI), tuberculosis (TB) is a highly prevalent infectious disease that continues to pose a serious challenge to public health. Patients with CFS and post-TB experience similar symptoms, such as fatigue, lassitude, and articular symptoms. TB arthritis commonly presents with chronic joint pain and insidious onset of only minimal signs of inflammation. Moreover, 72% of the patients with TB have moderate-to-severe anxiety and depression according to Hospital Anxiety and Depression Scale (HADS) [12], which considers some of the somatic symptoms. In addition, patients with CFS or with TB share similar plasma immunosignatures. Cytokine alterations are correlated with duration of illness, suggesting that CFS immunopathology is “not static” [13]. Abnormal cytokine profiles such as increased production of interferon (IFN) γ were observed in patients with CFS [14] and latent MTI [15]. Other immune activation markers of CFS include higher levels of the proinflammatory cytokines, tumour necrosis factor (TNF) α, interleukin (IL) 6, and IL-1β. Thus, the chronical activation and high dysregulation of the immune system may play an essential role in CFS development [16]. TB is chronic and remains latent in the human body for a lifetime. In this study, we investigated the association of TB and CFS by using retrospective cohort data from Taiwan National Health Insurance (NHI) Research Database (NHIRD).

## Methods

### Data source

We obtained our data from the Longitudinal Health Insurance Database 2000 (LHID2000) of NHIRD in this population-based retrospective cohort study. NHIRD contains all the reimbursement claims data from the NHI programme, including a beneficiary registry, medical records, a drug prescription registry, and other medical services. The programme is a nationwide single-payer insurance system established in March 1995 covering approximately 99% of Taiwanese residents [17]. LHID2000 contains registration and claims data of 1,000,000 insurants randomly sampled from the 2000 registry of NHIRD beneficiaries. The database renewed the claims data annually. The definition of disease in NHIRD is based on the International Classification of Diseases, Ninth Revision, Clinical Modification (ICD-9-CM). Before releasing LHID2000 for research, the original identification numbers of each insurant are removed through scrambling and linking individual claims data with random numbers.

This study was approved by the Research Ethics Committee of the China Medical University Hospital (CMUH-104-REC2-115) and the Institutional Review Board of Mackay Memorial Hospital (16MMHIS074).

### Participants

For the study cohort, we identified patients from LHID2000 aged ≥ 20 years and newly diagnosed as having a MTI (ICD-9-CM 010–018) between 2000 and 2011. The diagnosis date was defined as the index date. Patients aged below 20 years and patients with a history of CFS (ICD-9-CM 780.71) were excluded. The comparison cohort comprised individuals without MTI or CFS history. This cohort was randomly assigned the same index date as the MTI cohort. The comparison cohort was frequency-matched for age (strata of 5 years), sex, and index year at an approximately 4:1 ratio. All participants were followed from the index date until the date of CFS diagnosis, withdrawal from the programme, or the end of 2011, whichever was earliest.

### Comorbidities

Baseline comorbidity history of diabetes (ICD-9-CM 250), obesity (ICD-9-CM 278.0), renal disease (ICD-9-CM 580–589), rheumatoid arthritis (RA; ICD-9-CM 714), human immunodeficiency virus infection (ICD-9-CM 042), malignancy (ICD-9-CM 140–149, 150–159, 160–165, 170–172, 174–175, 179–189, 190–199, 200–208, and 235–238), and inflammatory bowel disease (IBD; ICD-9-CM 555–556) were obtained.

### Statistical analysis

The differences in demographic characteristics and comorbidities between the MTI and comparison cohorts were assessed using the chi-square test for categorical data and Student’s *t* test for continuous data. Cumulative incidence curves of CFS were computed using the Kaplan–Meier method, and between-cohort differences in cumulative incidence curves were assessed using the log-rank test. The incidence density of subsequent CFS for each cohort was calculated as the number of CFS events divided by the sum of follow-up duration (per 1000 person-years). Univariate and multivariate Cox proportional hazard regression models were used to examine the effect of MTI on CFS risk. The results are presented as hazard ratios (HRs) with 95% confidence intervals (CIs). The multivariate models were adjusted for age, sex, and comorbidities of diabetes, renal disease, and IBD. All analyses were generated using SAS (version 9.3; SAS Institute Inc., Cary, NC, USA), and a two-sided P value of < 0.05 was considered statistically significant.

## Results

**Fig. 1:**
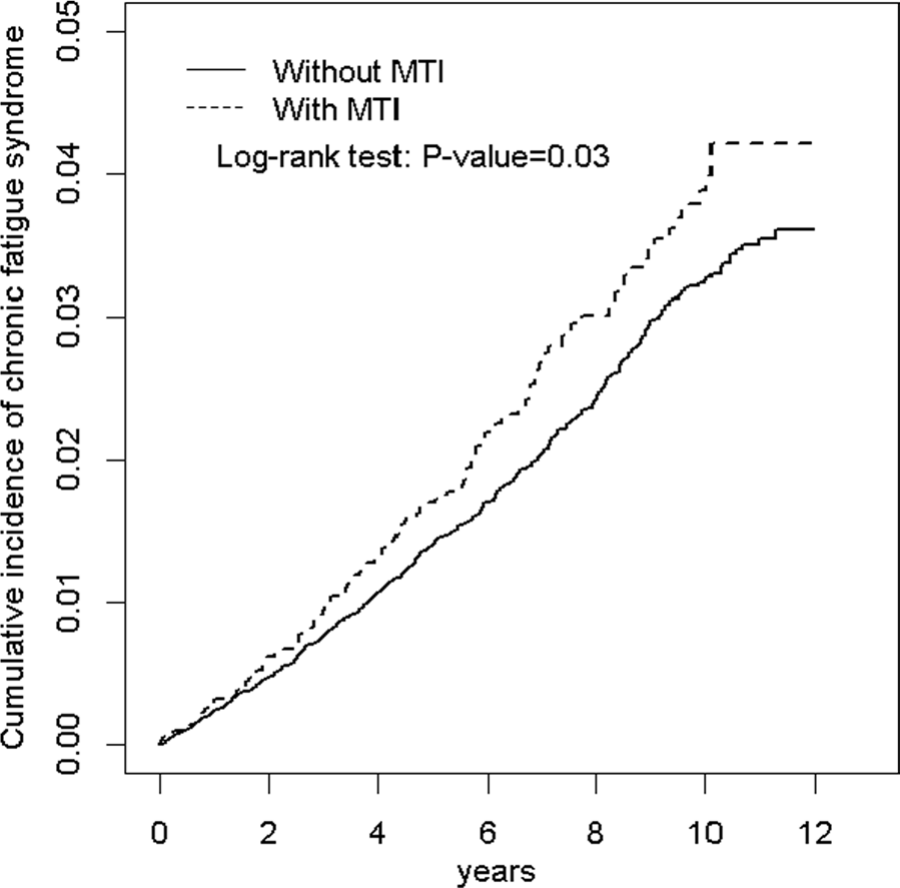
Cumulative incidence comparison of chronic fatigue syndrome for patients with (dashed line) or without (solid line) mycobacterium tuberculosis infection

The MTI and comparison cohorts comprised 7666 and 30,663 individuals, respectively, with similar age and sex distribution (Table 1). In the MTI cohort, 47. 8% of the participants were aged ≥ 65 years and 67.9% were men. In the MTI and comparison cohorts, the mean age was 60.5 ± 18.3 and 60.0 ± 18.3 years, respectively. Compared with the comparison cohort, the MTI cohort had significantly higher percentages of comorbidities except for obesity (all P < 0.05). In the MTI and comparison cohorts, the mean follow-up duration was 5.24 and 6.08 years, respectively. Figure 1 demonstrates that the cumulative incidence of CFS was significantly higher in the MTI cohort than in the comparison cohort (P = 0.03). In the MTI and comparison cohorts, the mean CFS incidence was 3.04 and 3.69 per 1000 person-years, respectively (Table 2). The multivariable models were mutually adjusting for age, sex, and comorbidities of diabetes, renal disease, and IBD.

**Table 1:** Demographic characteristics and comorbidities in cohorts with and without mycobacterium tuberculosis infection patients

Variable	Mycobacterium tuberculosis infection		*p*-value
	No	Yes	
	N = 30,663	N = 7666	
Age, year			0.99
≤ 34	3668 (12.0)	917 (12.0)	
35–49	5356 (17.5)	1339 (17.5)	
50–64	6992 (22.8)	1748 (22.8)	
65+	14,647 (47.8)	3662 (47.8)	
Mean ± SD^†^	60.0 (18.3)	60.5 (18.3)	0.01
Sex			0.99
Female	9836 (32.1)	2459 (32.1)	
Male	20,827 (67.9)	5207 (67.9)	
Comorbidity			
Diabetes	3473 (11.3)	1413 (18.4)	< 0.001
Obesity	294 (0.96)	50 (0.65)	0.01
Renal disease	2869 (9.36)	1061 (13.8)	< 0.001
Rheumatoid arthritis	43 (0.14)	30 (0.39)	< 0.001
HIV	13 (0.04)	34 (0.44)	< 0.001
Malignancy	1024 (3.34)	385 (5.02)	< 0.001
Inflammatory bowel disease	306 (1.00)	99 (1.29)	0.02

Chi-Square Test; ^†^: T-Test

**Table 2:** Incidence and Hazard ratio for chronic fatigue syndrome and chronic fatigue syndrome-associated risk factors

Variable	Event	PY	Rate^#^	Crude HR (95% CI)	Adjusted HR^&^ (95% CI)
Mycobacterium tuberculosis infection					
No	567	186,546	3.04	1.00	1.00
Yes	148	40,159	3.69	1.46 (1.04, 2.04)*	1.23 (1.03, 1.47)*
Age, year					
≤ 34	50	31,553	1.58	1.00	1.00
35–49	104	45,185	2.30	1.46 (1.04, 2.04)*	1.44 (1.02, 2.01)*
50–64	174	55,869	3.11	1.99 (1.45, 2.72)***	1.90 (1.38, 1.60)***
65 +	387	94,097	4.11	2.71 (2.01, 3.63)***	2.50 (1.86, 3.38)***
Sex					
Female	227	75,147	3.02	1.00	1.00
Male	488	151,558	3.22	1.07 (0.92, 1.25)	–
Comorbidity					
Diabetes					
No	610	203,033	3.00	1.00	1.00
Yes	105	23,672	4.44	1.53 (1.24, 1.88)***	1.20 (0.97, 1.49)
Obesity					
No	710	224,949	3.16	1.00	1.00
Yes	5	1756	2.85	0.93 (0.39, 2.24)	–
Renal disease					
No	618	207,626	2.98	1.00	1.00
Yes	97	19,079	5.08	1.76 (1.42, 2.18)***	1.41 (1.13, 1.76)**
Rheumatoid arthritis					
No	715	226,313	3.16	1.00	1.00
Yes	0	392	0.00	–	–
HIV					
No	714	226,474	3.15	1.00	1.00
Yes	1	231	4.32	1.41 (0.20, 10.0)	–
Malignancy					
No	693	221,075	3.13	1.00	1.00
Yes	22	5630	3.91	1.30 (0.85, 1.99)	–
Inflammatory bowel disease					
No	702	224,623	3.13	1.00	1.00
Yes	13	2082	6.24	2.05 (1.19, 3.55)*	1.83 (1.06, 3.17)*

Rate^#^, incidence rate, per 1000 person-years; Crude HR *, relative hazard ratio; Adjusted HR^†^: multivariable analysis including age, sex and comorbidities of diabetes, renal disease, and inflammatory bowel disease

*p < 0.05, **p < 0.01, ***p < 0.001

**Fig. 2:**
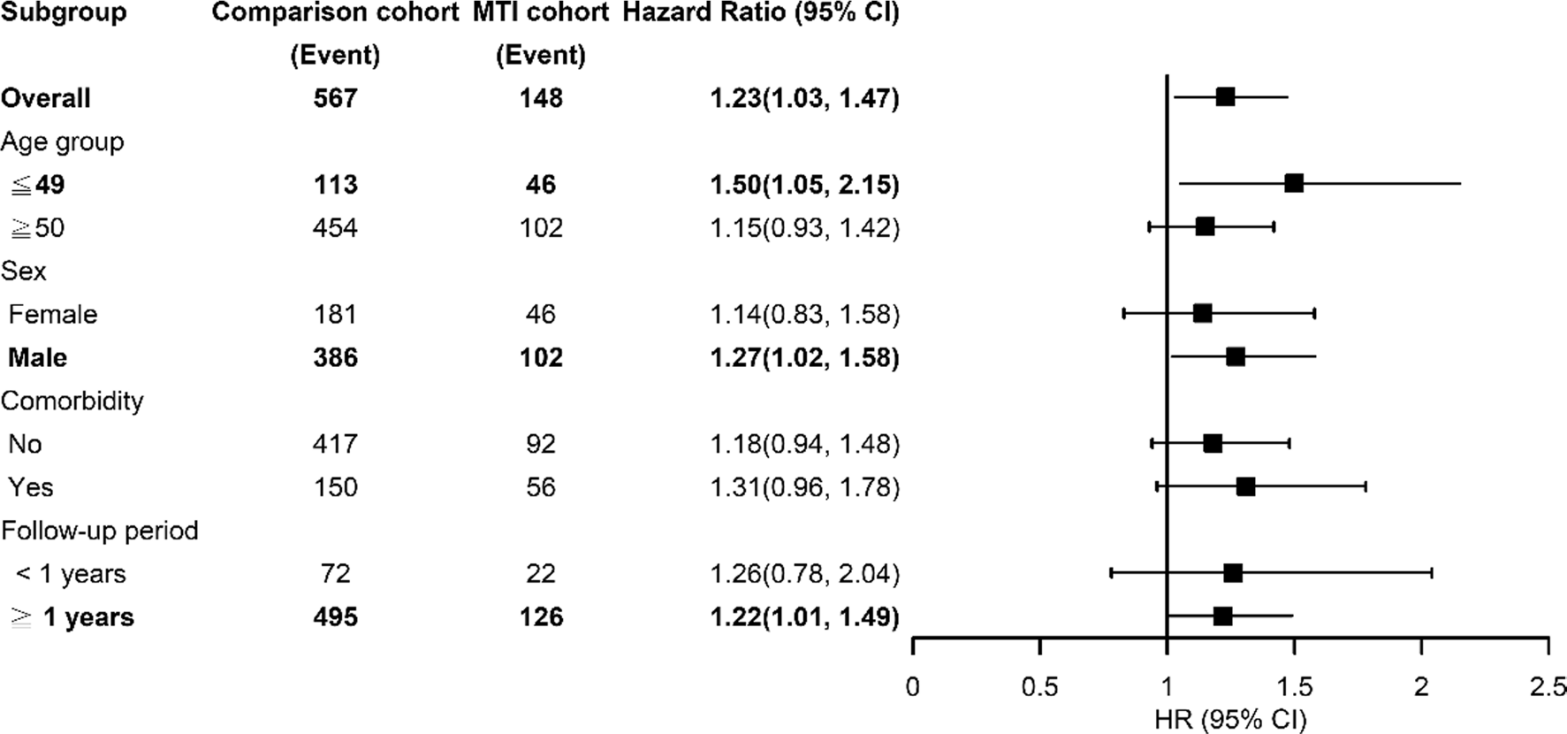
Events of chronic fatigue syndrome by age, sex and comorbidity and Cox model measured hazards ratio and bar charts for patients with mycobacterium tuberculosis infection compared those without mycobacterium tuberculosis infection

After mutually adjusted for age, sex, and comorbidities of diabetes, renal disease, and IBD, the risk of CFS had a 1.23-fold greater in the MTI cohort than in the comparison cohort (95% CI = 1.03–1.47). After mutually adjusting for CFS, sex, and comorbidities of diabetes, renal disease, and IBD, compared with patients aged 34 years and younger, the risk of CFS development is 2.50-fold higher in those aged 65 and more than 65 years (95% CI = 1.86–3.38), 1.90-fold higher in those aged 50–64 years and 1.44-fold higher in those aged 35–49 years (95% CI = 1.02–2.01). Patients with renal disease and IBD had 1.41 (95% CI = 1.13–1.76) and 1.83 (95% CI = 1.06–3.17) times higher CFS risk, respectively. Compared with the participants without MTI, participants aged ≤ 49 years in the MTI cohort had 1.5 (95% CI = 1.05–2.15) times higher CFS risk (Table 3, Fig. 2). Men had 1.27 (95% CI = 1.02–1.58) times higher CFS risk in the MTI cohort than in the comparison cohort. After > 1 year of follow-up, CFS risk remained 1.22 (95% CI = 1.01–1.49) times higher in the MTI cohort than in the comparison cohort. Table 4 presents the data on the effects of CFS-associated comorbidities on CFS risk. The data showed that compared with participants without either condition, participants with MTI and diabetes disease had 1.61 (95% CI = 1.12–2.31) times increased CFS risk.

**Table 3:** Incidence of chronic fatigue syndrome by age, sex and comorbidity and Cox model measured hazards ratio for patients with mycobacterium tuberculosis infection compared those without mycobacterium tuberculosis infection

Variables	Mycobacterium tuberculosis infection						Crude HR^*^ (95% CI)	Adjusted HR^&^ (95% CI)
	No			Yes				
	Event	PY	Rate^#^	Event	PY	Rate^#^		
Age, years								
≤ 49	113	62,036	1.82	41	14,703	2.79	1.54 (1.08, 2.20)*	1.50 (1.05, 2.15)*
≥ 50	454	124,510	3.65	107	25,456	4.20	1.17 (0.95, 1.45)	1.15 (0.93, 1.42)
Sex								
Female	181	61,227	2.96	46	13,920	3.30	1.12 (0.81, 1.55)	1.14 (0.83, 1.58)
Male	386	125,319	3.08	102	26,239	3.89	1.28 (1.03, 1.59)*	1.27 (1.02, 1.58)*
Comorbidity								
No	417	152,391	2.74	92	29,475	3.12	1.15 (0.92, 1.44)	1.18 (0.94, 1.48)
Yes	150	34,155	4.39	56	10,684	5.24	1.20 (0.88, 1.63)	1.31 (0.96, 1.78)
Follow-up period								
< 1 years	72	30,136	2.39	22	7156	3.07	1.29 (0.80, 2.08)	1.26 (0.78, 2.04)
> 1 years	495	156,410	3.16	126	33,002	3.82	1.21 (1.00, 1.48)*	1.22 (1.01, 1.49)*

Rate^#^, incidence rate, per 1000 person-years; Crude HR *, relative hazard ratio; Adjusted HR^†^: multivariable analysis including age, sex, and comorbidities of diabetes, renal disease, and inflammatory bowel disease;

^*^p < 0.05, **p < 0.01, ***p < 0.001

**Table 4:** Cox Proportional Hazard Regression Analysis for the risk of chronic fatigue syndrome-associated mycobacterium tuberculosis infection with joint effects of comorbidities

Variables		N	Event	Adjusted HR^‡^ (95% CI)
Mycobacterium tuberculosis infection	Diabetes			
No	No	27,190	494	1 (Reference)
No	Yes	3473	73	1.16 (0.90, 1.49)
Yes	No	6253	116	1.19 (0.98, 1.46)
Yes	Yes	1413	32	1.61 (1.12, 2.31)**
Mycobacterium tuberculosis infection	Renal disease			
No	No	27,794	494	1 (Reference)
No	Yes	2869	73	1.39 (1.08, 1.78)*
Yes	No	6605	124	1.23 (1.01, 1.50)*
Yes	Yes	1061	24	1.69 (1.12, 2.56)*
Mycobacterium tuberculosis infection	Inflammatory bowel disease			
No	No	30,357	557	1 (Reference)
No	Yes	306	10	1.83 (0.98, 3.43)
Yes	No	7567	145	1.23 (1.02, 1.48)*
Yes	Yes	99	3	2.13 (0.69, 6.60)

## Discussion

The current results indicated that had a significantly higher CFS incidence in the MTI cohort than in the comparison cohort. The subgroup analysis demonstrated that male patients and those aged ≤ 49 years in the MTI cohort had a relatively high HR for CFS. This finding has not been reported previously. In addition, patients with MTI who had CFS-associated comorbidities such as renal disease and diabetes had increased CFS risk.

Our results suggest that men with MTI are more likely to be diagnosed as having CFS. In developed countries, MTI incidence is higher in older individuals than in younger individuals, and it is higher in men than in women [18, 19]. These findings are consistent with those in the present study (Table 1). A meta-analysis reported that older patients with pulmonary TB have lower rates of fever and sweating and lower leukocyte concentrations [20]. The current results suggest that patients with MTI aged ≤ 49 years have an increased CFS risk (Table 3), possibly because of differences in immune response between older and younger individuals [21]. However, these relevant mechanisms and immunomodulating effects of aging require further investigation.

CFS is a multifactorial disease caused by pathogens, including the Epstein–Barr virus, human herpes virus 6, and human parvovirus B19 [22, 23]. Our current findings suggest that TB is correlated with CFS. Studies have proposed possible mechanisms of disease, including immunoinflammatory pathways [14, 16], neuroimmune dysfunction [16], oxidative and nitrosative stress (O&NS) pathways [24, 25], and bacterial translocation [26].

Immunoinflammatory pathway activation is one of the most researched topics related to CFS [14, 16]. Immune activation markers in CFS include increased levels of proinflammatory cytokines such as TNF-α, IL-6, and IL-1β [27, 28]. In patients with TB, the interaction of *M. tuberculosis* ligands with Toll-like receptors eventually results in immune activation, including activated nuclear factor (NF) κB and TNF-α, IL-1, and IL-12 production through myeloid differentiation primary response protein 88-dependent or -independent pathways [29, 30]. Increased production of NF-κB, a major upstream molecule regulating immunoinflammatory response, is associated with fatigue and a subjective feeling of infection [31].

TNF-α, which is secreted by macrophages, dendritic cells, and T cells, plays a major protective role against MTI and transmits signals regulating immune cell migration to the infection sites [32] and the formation of microbicidal granulomas [33]. IL-1 and TNF-α levels are significantly positively correlated with fatigue, autonomic symptoms, and flu-like symptoms [34]. TNF-α inhibitors, a type of immunomodulator, has also been reported to alleviate fatigue symptoms in some autoimmune diseases [35, 36] and attenuate CFS risk in patients with psoriasis [37].

IFN-γ, which is produced by activated T cells and natural killer cells activated by peripheral macrophages [38], appears in patients with latent MTI and plays a critical defensive role against MTI. IFN-γ synergizes with TNF-α and activates macrophages to kill intracellular bacilli [33]. The production of Th1 cytokines such as IFN-γ in patients with CFS is associated with the extent of fatigue [39]. A recent study observed that individuals with specific genetic polymorphisms of IFN-γ experience more severe fatigue as part of an acute postinfectious sickness [40]. IFN-γ-mediated lesions in kynurenine metabolism may culminate in depression and psychomotor retardation and contribute to disability in some patients with CFS [13].

Increased numbers of reactive oxygen and nitrogen species and activated O&NS pathways may be involved in CFS pathogenesis [24, 25]. This hypothesis is based on reports of increased production of inducible nitric oxide synthase (iNOS) [31] and reduced levels of antioxidants [16]. Macrophages activated by IFN-γ and TNF-α [41] produce nitric oxide and other reactive nitrogen species through iNOS to exert toxic effects on *M. tuberculosis*. iNOS activity inhibition leads to latent MTI reactivation in mice [41]. Low-grade inflammation, activated O&NS pathways, and impaired oxidative defenses in CFS potentially interact to increase the magnitude of abnormality in each, constituting a vicious cycle [14]. Lactate, an antioxidant that scavenges free radicals and attenuates lipid peroxidation [42], may explain the reason that in patients with CFS, fatigue was significantly reduced and functional capacity and fitness function were significantly improved after exercise treatment compared with after flexibility treatment [43].

Pathogens commonly associated with CFS are capable of establishing prolonged infection as a result of developing sophisticated adaptations to the host immune response [16]. For example, the varicella zoster virus [44] migrates along sensory axons to establish latency in neurons within the regional ganglia and only expresses a limited number of viral proteins [45] Similarly, *M. tuberculosis* has numerous defensive mechanisms to circumvent host immunity, such as disrupting the maturation of bacilli-containing phagosomes into phagolysosomes through the exclusion of vH^+^-ATPase during phagosomal maturation to prevent their destruction by lysosomal enzymes. Although only 10% of the patients with MTI develop TB, *M. tuberculosis* will remain in the nonreplicating state within granuloma in the other 90%. A disturbance of the immune system (e.g., old age, malnutrition, or medical conditions [46]), can trigger TB development. Despite reactivation of latent TB, inflammation can occur when *M. tuberculosis* spreads to a new location through aerosols generated by inspired air because foamy macrophages phagocytose extracellular nonreplicating *M. tuberculosis*, drain from lung granuloma toward the bronchial tree, and return to a different region of lung parenchyma through air inspiration [47]. These new infection sites may attract immune cells, which induce all the characteristic symptoms of CFS. These reactivation-resolution and migration cycles in TB lead to the mentioned inflammatory responses that may explain the chronic and relapsing–remitting nature of CFS.

A study found administration of the antituberculosis agent, isoniazid (300 mg per day for 30 days) to alleviate CFS symptoms, as demonstrated through improved Multidimensional Fatigue Inventory and the Zung Depression Scale scores [48]. However, the effect was not long-lasting; after 6 months, the TB was reactivated. Patients with latent TB should receive antibiotic therapy with a longer treatment course to prevent TB activation [49].

Our study has several limitations. As our previous study of LHID [50, 51], data on patient history, including symptoms, occupation status, contact history, and disease severity, are unavailable in NHIRD. Furthermore, the study population was mainly composed of East Asian people living in Taiwan, which limits the generalizability of the findings to other ethnicities. Although minor database errors in diagnostic coding can affect the data analysis results, such biases result in considerable penalties for physicians who have been more meticulous when recording codes. In addition, NHIRD enrolls 99.9% of Taiwan’s population, and its reliability and validity for epidemiological investigations have been reported previously [52, 53]. Therefore, the diagnostic coding used in the present study should be reliable.

In conclusion, this is a first paper to prove the novel findings about the association of MTI and CFS. They have common immunoinflammatory pathway and cytokines such as TNF-α, IL-1, IL-6, IFN-γ and NF-κB pathway. In addition, *M. tuberculosis* has numerous defensive mechanisms and are capable of intracellular persistence to circumvent host Immunity [54]. Although we didn’t explore the direct causality between MTI and CFS, we provide new perceptions for future studies to evaluate the actual mechanisms.

## Conclusion

This study is the first population-based study to investigate the risk of CFS in patients with MTI, and its pilot finding is sufficient to provide perceptions for recognizing high-risk people likely to suffer from CFS. Future studies could examine the mechanisms underlying CFS risk following tuberculosis and discover the preventive and personalised medicine to improve the patient’s quality of life.

## Data Availability

The data underlying this study is from the National Health Insurance Research database (NHIRD). Interested researchers can obtain the data through formal application to the Ministry of Health and Welfare, Taiwan.

## Abbreviations

CFS: Chronic fatigue syndrome
TB: Tuberculosis
MTI: Mycobacterium tuberculosis infection
HADS: Hospital Anxiety and Depression Scale
IFN: Interferon
TNF: Tumour necrosis factor
IL: Interleukin
NHIRD: National Health Insurance Research Database
NF: Nuclear factor
